# Associations among amino acid, lipid, and glucose metabolic profiles in childhood obesity

**DOI:** 10.1186/s12887-019-1647-8

**Published:** 2019-08-06

**Authors:** Yosuke Suzuki, Jun Kido, Shirou Matsumoto, Kie Shimizu, Kimitoshi Nakamura

**Affiliations:** 1 Department of Pediatrics, Graduate School of Medical Sciences, Kumamoto University, 1-1-1 Honjo, Kumamoto City, Kumamoto Prefecture 860-8556 Japan; 2 Department of Central Radiology, Kumamoto University Hospital, Kumamoto University, Kumamoto City, Kumamoto Japan

**Keywords:** Amino acids, Homeostasis model assessment-insulin resistance, Obesity, Uric acid

## Abstract

**Background:**

Plasma-free amino acid profiles have been reported to correlate with obesity and glucose metabolism, and have been studied as potentially useful biomarkers of lifestyle-related diseases affecting metabolism in adulthood. However, knowledge of these relationships is lacking in children, despite the growing public health problem posed by childhood obesity.

The aim of this study was to assess whether plasma-free amino acid profiles can serve as useful biomarkers of lifestyle-related diseases in children with obesity.

**Methods:**

This retrospective study used the medical records of 26 patients (15 male, 11 female) aged 9 or 10 years presenting with moderate to severe obesity and hyperlipidemia between April 2015 and March 2017. A degree of obesity of 30% or more was defined as moderate or severe. Amino acid levels were compared between obese children with and without impaired glucose tolerance using a t-test or Mann–Whitney U test. In addition, the influence of factors such as intima media thickness, low-density lipoprotein cholesterol, high-density lipoprotein cholesterol, amino acids, and homeostasis model assessment-insulin resistance (HOMA-IR) were analyzed pairwise using Pearson’s correlation or Spearman’s rank correlation.

**Results:**

HOMA-IR was positively correlated with valine, leucine (Leu), isoleucine, phenylalanine, tryptophan, methionine, threonine, lysine, alanine, tyrosine, glutamate (Glu), proline, arginine, ornithine, total free amino acids (all *P* < 0.01), and aspartate (*P* = 0.010). Moreover, blood uric acid levels were positively correlated with Leu (*P* = 0.005) and Glu (*P* = 0.019), and negatively correlated with serine, glycine, and asparagine (*P* = 0.007, *P* = 0.003, and *P* = 0.013, respectively).

**Conclusions:**

Amino acid profile reflects impaired glucose tolerance and hyperuricemia at an early stage of obesity. It is therefore a useful marker to inform early intervention in children with obesity, as in adults.

**Electronic supplementary material:**

The online version of this article (10.1186/s12887-019-1647-8) contains supplementary material, which is available to authorized users.

## Background

Childhood obesity is one of the most serious public health problems. The number of obese children under the age of five is gradually increasing all over the world. Forty-two million children under 5 years of age are estimated to be affected by overweightness and obesity worldwide [1]. Overweightness and obesity in early childhood also lead to a higher risk of overweightness and obesity in adulthood [2], and confer an increased risk of chronic inflammatory conditions including diabetes mellitus (DM), cardiovascular diseases, non-alcoholic fatty liver disease, and some cancers. Although cerebrovascular and cardiovascular events rarely occur in childhood, even in severe obesity, obesity in childhood is likely to result in a significant long-term economic burden on society, with associated excess lifetime health care [3] and indirect costs [4] including sick leave, reduced productivity, and premature mortality. Symptomatic pediatric lifestyle-related diseases are present in 5–15% of obese children and their incidence increases after late elementary school age. Moreover, obesity in childhood drives higher morbidity and mortality compared to obesity developed during adulthood. Therefore, intervention against childhood obesity is very important. This study focused on children aged 9 or 10 years who were subjected to screening and intervention for obesity to prevent adult and pediatric lifestyle diseases.

Amino acid (AA) profiles have been used as a biomarker of obesity and DM. We previously reported that the plasma concentrations of valine (Val), leucine (Leu), and isoleucine (Ile), as well as the total branched chain amino acids (BCAA), alanine (Ala), citrulline (Cit), and proline (Pro), were significantly higher in diabetic mice than in normal mice [5]. Wang et al. [6] reported a 12-year follow-up study showing that plasma levels of BCAA, tyrosine (Tyr), and phenylalanine (Phe) could be predictors of the future development of diabetes in nondiabetic subjects. Other studies have reported significant associations between the plasma levels of specific AAs and body mass index (BMI) [7], AAs, and glucose regulation [8]. In a study on Japanese obesity, Takashina et al. [9] reported specific associations between specific AAs including Val, Leu, Ala, and Cit, the type/degree of obesity, and indices of glucose/insulin regulation in Japanese adults with normal glucose metabolism.

In this study, we analyzed the correlation of blood amino acids and obese metabolic states to assess whether plasma-free amino acid profiles can become useful biomarkers of lifestyle-related diseases in children with obesity. Moreover, we discussed the metabolic role of amino acids in children with obesity. This study included a clinical laboratory-based examination and measurement of the intima media thickness (IMT) of the internal carotid artery as a marker of metabolic state.

## Methods

### Study design

We retrospectively studied the medical records of 26 patients (male: 15, female: 11), aged 9 or 10 years, who presented in the Department of Pediatrics, Kumamoto University Hospital with moderate to severe obesity (defined as a degree of obesity ≥30%) at the first and second (after 6 months) screenings performed in Kumamoto City between April 2014 and March 2016. Degree of obesity was calculated according to the formula: ([real body weight–standard body weight depending on age] ÷ the standard weight × 100), as defined by the Japanese Society for Pediatric Endocrinology [10].

### Clinical evaluation

Clinical information, including age, sex, symptoms, present condition, medical history, medication use, and family history, was recorded on a standardized data form by the examining medical staff during the patients’ visits. The degree of obesity, body mass index (BMI), blood pressure, blood uric acid (UA), liver function [alanine aminotransferase (ALT), aspartate aminotransferase (AST), lactate dehydrogenase (LDH), and γ-glutamyltransferase (γ-GTP)], glucose tolerance [fasting blood glucose, insulin, C-peptide, and homeostasis model assessment-insulin resistance (HOMA – IR)], and blood lipid levels [total cholesterol (T-CHO), low-density lipoprotein cholesterol (LDL-CHO), high-density lipoprotein cholesterol (HDL-CHO), and triglyceride (TG)] were evaluated. The blood samples of obese children were collected after fasting for 12 h.

### Assay of amino acid levels and measurement of intima media thickness

Plasma amino acids were analyzed using a liquid chromatograph mass spectrometer (SRL, Inc., Tokyo, Japan). The IMTs of the internal left and right carotid arteries were measured using an Aplio XG ultrasound machine (Toshiba Medical System Corporation, Tochigi, Japan) and double-checked by two technicians. The IMTs were measured at three points of both internal carotid arteries and averaged (Additional file 1).

### Data quality analysis

Two researchers, who did not participate in the medical diagnosis, ultrasonography, blood analysis, or medical record evaluation, performed the data and statistical analyses in this study.

### Statistical analysis

We compared amino acid levels between obese children with and without impaired glucose tolerance using a t-test or Mann–Whitney U test. The factors IMT, LDL- and HDL-CHO (LDL/HDL ratio), amino acids, HOMA-IR, and UA were analyzed pairwise using Pearson’s correlation or Spearman’s rank correlation in IBM SPSS Statistics ver. 25. HOMA-IR and UA were dependent variables for predicting blood amino acid values, such as Val, Leu, and Ile, which are independent variables. The IMT and LDL/HDL ratio, were not dependent variables for blood amino acid values. A two-sided probability value of *P* < 0.05 was considered to be statistically significant. We also compared BMI, IMT, insulin, LDL-CHO, and amino acid levels before and after intervention using a paired t-test or Wilcoxon signed-rank test. Average values are presented as the mean ± one standard deviation.

### Ethical approval and informed consent

This study was approved by the Ethics Committee of the Graduate School of Medical Sciences, Kumamoto University. Informed consent was obtained from the parents of all the children included in this study or the parents and children.

## Results

### Clinical observations

In this study, we evaluated 15 male (age: 122.2 ± 4.2 months) and 11 female (age: 122.9 ± 4.1 months) obese children. Their heights, weights, and BMIs were 140.4 ± 6.4 and 140.0 ± 4.2 cm, 46.6 ± 7.6 and 45.8 ± 6.9 kg, and 23.5 ± 2.5 and 23.3 ± 2.4, respectively (Additional file 1).

Nineteen percent (5/26) of obese children in this study developed simple obesity with no abnormalities in blood data, 58% (15/26) developed hypercholesterolemia (LDL-CHO ≥ 140 mg/dL), 19% (5/26) developed hypertriglyceridemia (TG ≥ 120 mg/dL), 8% (2/26) developed hypoHDLemia (HDL-CHO < 40 mg/dL), 19% (5/26) developed hyperuricemia (UA ≥ 6 mg/dL), 35% (9/26) developed impaired glucose tolerance (HOMA-R ≥ 2.5), and 27% (7/26) developed liver damage (ALT > 30 IU/L). None of the children presented significant arteriosclerotic lesions in either of their internal carotid arteries. The average IMTs were 0.54 ± 0.06 mm (left: 0.55 ± 0.07 mm; right: 0.54 ± 0.07 mm).

### Correlations involving lipid levels

T-CHO and LDL-CHO levels were negatively correlated with BMI, HOMA-IR, blood insulin, and γ-GTP levels (Table 1). Levels of HDL were negatively correlated with IMT (*N* = 26; *P* = 0.039), and LDL/HDL ratios were positively correlated with IMT (*N* = 26; *P* = 0.023) (Table 1). We also observed a negative correlation between the LDL/HDL ratio and blood Tyr levels. Additional file 1 details comparisons of laboratory metabolic data between obese children with LDL/HDL ratio ≤ 2.0 and LDL/HDL ratio > 2.0. There were no significant associations between LDL/HDL ratio and HOMA-IR or LDL/HDL ratio and amino acids (Tables 1 and 2, Additional file 1). However, LDL/HDL ratio was positively correlated with IMT (Additional file 1).

**Table 1:**
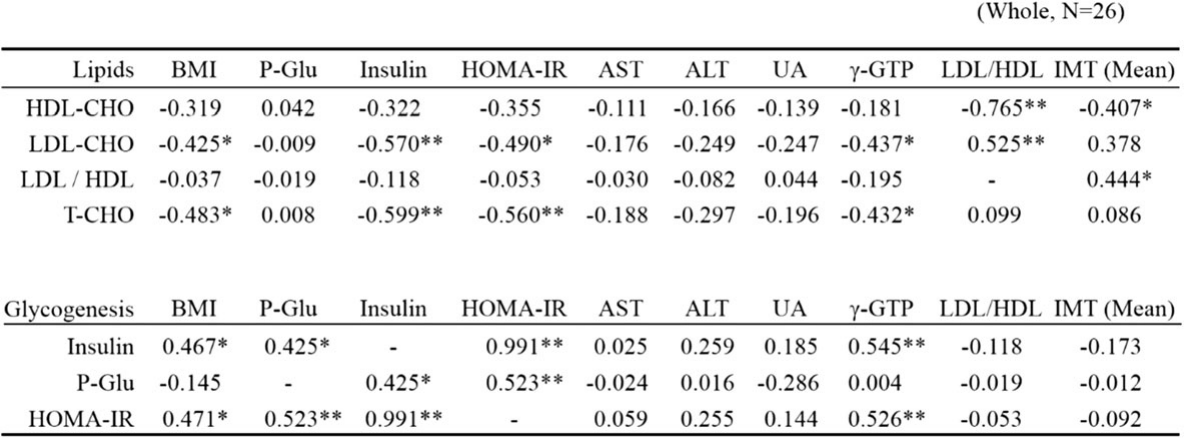
Correlation of physical and biochemical variables in children with obesity

*BMI* body mass index, *P-Glu* plasma glucose, *AST* aspartate aminotransferase, *ALT* alanine aminotransferase, *UA* Uric acid, *γ-GTP* γ-glutamyltransferase, *IMT* intima media thickness, *HDL* high-density lipoprotein, *LDL* low-density lipoprotein, *TCHO* total cholesterol. *N* = 26; *P < 0.05; **P < 0.01

**Table 2:**
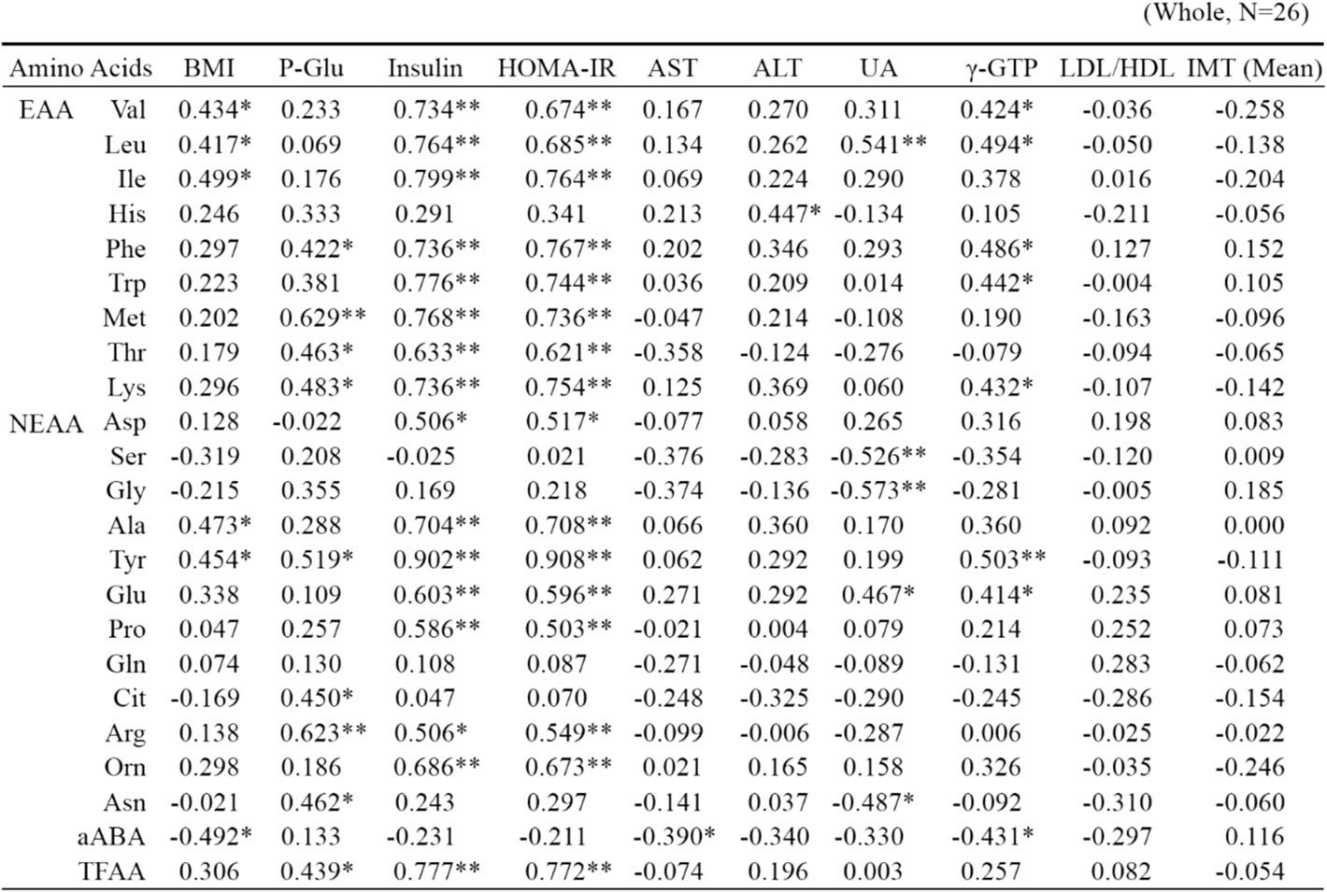
Plasma amino acids profile in children with obesity

*BMI* body mass index, *P-Glu* plasma glucose, *AST* aspartate aminotransferase, *ALT* alanine aminotransferase, *UA* uric acid, *γ-GTP* γ-glutamyltransferase, *HDL* high-density lipoprotein, *LDL* low-density lipoprotein, *IMT* intima media thickness, *EAA* essential amino acids, *Val* Valine, *Leu* leucine, *Ile* isoleucine, *His* histidine, *Phe* phenylalanine, *Trp* tryptophan, *Met* methionine, *Thr* threonine, *Lys* lysine, *NEAA* non-essential amino acids, *Asp* aspartate, *Ser* Serine, *Gly* glycine, *Ala* alanine, *Tyr* tyrosine, *Glu* glutamate, *Pro* proline, *Gln* glutamine, *Cit* citrulline, *Arg* arginine, *Orn* ornithine, *Asn* asparagine, *aABA* α-aminobutyric acid, *TAA* total amino acids. *N* = 26; *P < 0.05; **P < 0.01

### Correlations involving insulin resistance

**Fig. 1:**
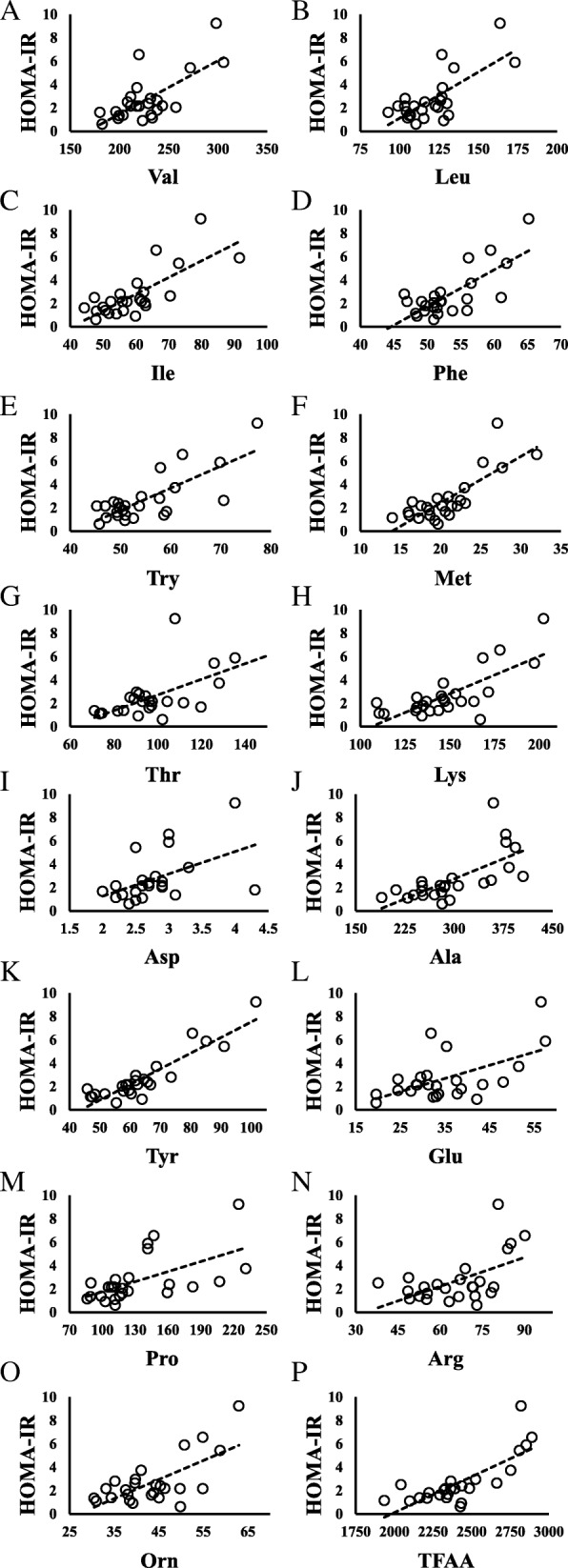
Relationship between HOMA-IR and amino acids in children with impaired glucose tolerance (HOMA-IR ≥2.5). **a** HOMA-IR vs Valine; *N* = 9, y = 0.0434x - 5.9926, *R*^*2*^ = 0.5007, *P* = 0.033. **b** HOMA-IR vs Leucine; *N* = 9, y = 0.0776x - 5.6865, *R*^*2*^ = 0.5482, *P* = 0.023. **c** HOMA-IR vs Phenylalanine; *N* = 9, y = 0.2707x - 10.711, *R*^*2*^ = 0.4843, *P* = 0.037. **d** HOMA-IR vs Tryptophan; *N* = 9, y = 0.1725x - 6.0928, *R*^*2*^ = 0.4479, *P* = 0.049. **e** HOMA-IR vs Methionine; *N* = 9, y = 0.3807x - 4.4448, *R*^*2*^ = 0.5995, *P* = 0.014. **f** HOMA-IR vs Lysine; *N* = 9, y = 0.0787x - 8.4333, *R*^*2*^ = 0.6733, *P* = 0.007. **g** HOMA-IR vs Tyrosine; *N* = 9, y = 0.151x - 6.9265, *R*^*2*^ = 0.843, *P* = 0.000. **h** HOMA-IR vs Arginine; *N* = 9, y = 0.0899x - 1.7275, *R*^*2*^ = 0.4625, *P* = 0.044. **i** HOMA-IR vs Ornithine; *N* = 9, y = 0.2148x - 5.5718, *R*^*2*^ = 0.8164, *P* = 0.001. **j** HOMA-IR vs Total amino acids; *N* = 9, y = 0.0056x - 10.198, *R*^*2*^ = 0.4655, *P* = 0.043

Figure 1 shows scatter diagrams demonstrating correlations between HOMA-IR and amino acid levels. HOMA-IR was positively correlated with Val, Leu, Ile, Phe, tryptophan (Trp), methionine (Met), threonine (Thr), lysine (Lys), Ala, Tyr, glutamate (Glu), Pro, arginine (Arg), ornithine (Orn), and total free amino acids (TFAA) (all *P* < 0.01), and aspartate (Asp) (*P* = 0.010) (Table 2).

**Fig. 2:**
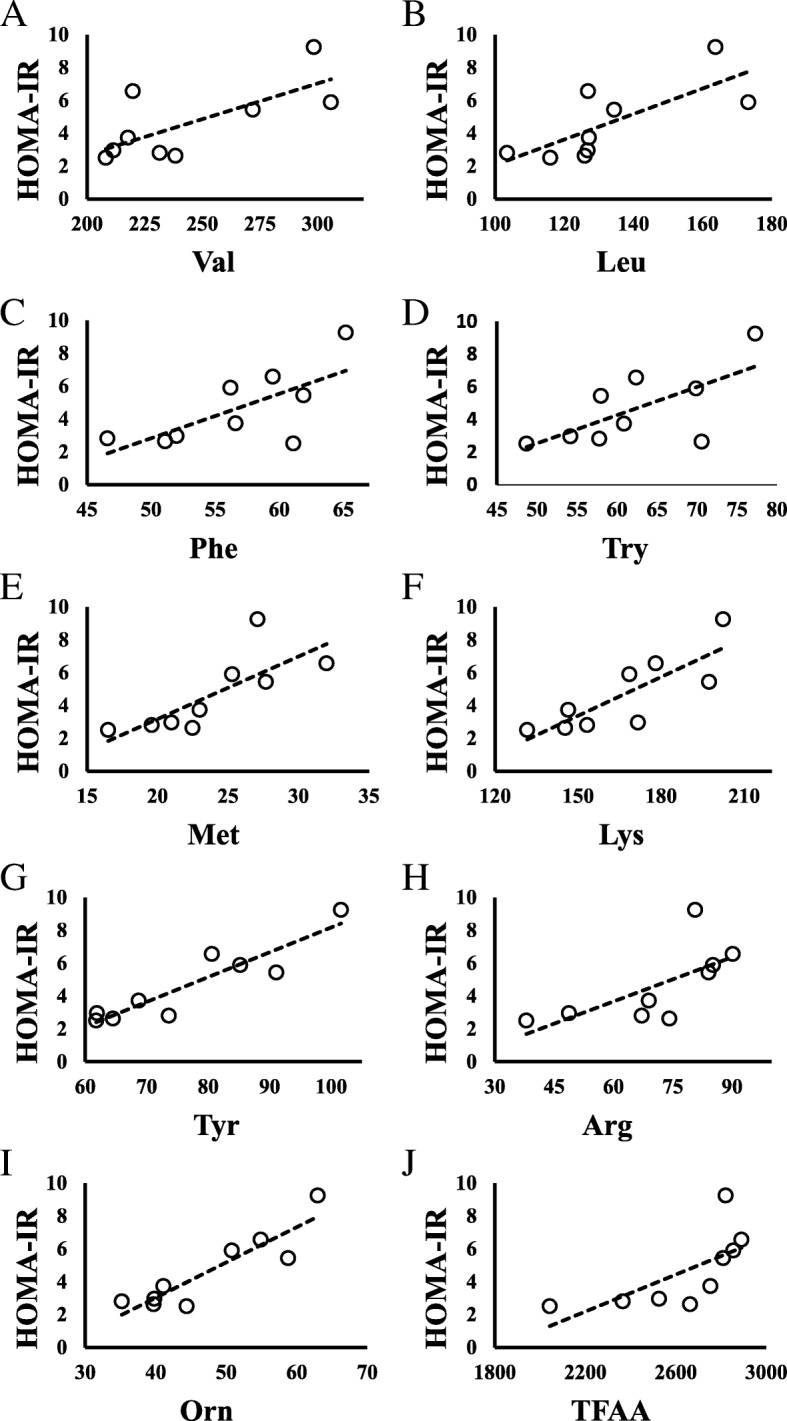
Relationship between HOMA-IR and amino acids in all children. **a** HOMA-IR vs Valine; *N* = 26, y = 0.0456x - 7.6445, *R*^*2*^ = 0.488, *P* = 0.000. **b** HOMA-IR vs Leucine; *N* = 26, y = 0.0791x - 6.7905, *R*^*2*^ = 0.5249, *P* = 0.000. **c** HOMA-IR vs Isoleucine; *N* = 26, y = 0.1423x - 5.7363, *R*^*2*^ = 0.5791, *P* = 0.000. **d** HOMA-IR vs Phenylalanine; *N* = 26, y = 0.3157x - 14.067, *R*^*2*^ = 0.5585, *P* = 0.000. **e** HOMA-IR vs Tryptophan; *N* = 26, y = 0.1893x - 7.6714, *R*^*2*^ = 0.5930, *P* = 0.000. **f** HOMA-IR vs Methionine; *N* = 26, y = 0.3979x - 5.5475, *R*^*2*^ = 0.6411, *P* = 0.000. **g** HOMA-IR vs Threonine; *N* = 26, y = 0.067x - 3.9788, *R*^*2*^ = 0.4209, *P* = 0.001. **h** HOMA-IR vs Lysine; *N* = 26, y = 0.0641x - 6.8073, *R*^*2*^ = 0.5611, *P* = 0.000. **i** HOMA-IR vs Aspartate; *N* = 26, y = 1.9114x - 2.5529, *R*^*2*^ = 0.2374, *P* = 0.010. **j** HOMA-IR vs Alanine; *N* = 26, y = 0.0228x - 4.105, *R*^*2*^ = 0.4683, *P* = 0.000. **k** HOMA-IR vs Tyrosine; *N* = 26, y = 0.1339x - 5.8458, *R*^*2*^ = 0.8182, *P* = 0.000. **l** HOMA-IR vs Glutamate; *N* = 26, y = 0.1127x - 1.2491, *R*^*2*^ = 0.3196, *P* = 0.001. **m** HOMA-IR vs Proline; *N* = 26, y = 0.0287x - 1.1086, *R*^*2*^ = 0.3333, *P* = 0.002. **n** HOMA-IR vs Arginine; *N* = 26, y = 0.0827x - 2.7473, *R*^*2*^ = 0.2986, *P* = 0.004. **o** HOMA-IR vs Ornithine; *N* = 26, y = 0.1631x - 4.394, *R*^*2*^ = 0.4691, *P* = 0.000. **p** HOMA-IR vs Total AA; *N* = 26, y = 0.0061x - 12.182, *R*^*2*^ = 0.6059, *P* = 0.000

Metabolic data from obese children with HOMA-IR ≤1.6, 1.6 < HOMA-IR < 2.5, and HOMA-IR ≥ 2.5 are presented in Tables 3 and 4. The blood levels of Val, Leu, Ile, Phe, Trp, Met, Thr, Lys, Asp, Ala, Tyr, Pro, Hydroroxyproline, TFAA, essential amino acids (EAA), non-essential amino acids (NEAA), and branched-chain amino acids (BCAA) were higher in obese children with impaired glucose tolerance (HOMA-IR ≥ 2.5, *N* = 8) than in those without impaired glucose tolerance (HOMA -IR ≤1.6, *N* = 9) (Table 4). Children with impaired glucose tolerance (HOMA-IR ≥2.5) showed positive correlations between HOMA-IR and levels of Val, Leu, Phe, Trp, Met, Lys, Tyr, Arg, Orn, and TFAA. Figure 2 shows scatter diagrams demonstrating correlations between HOMA-IR and blood amino acid levels.

**Table 3:**
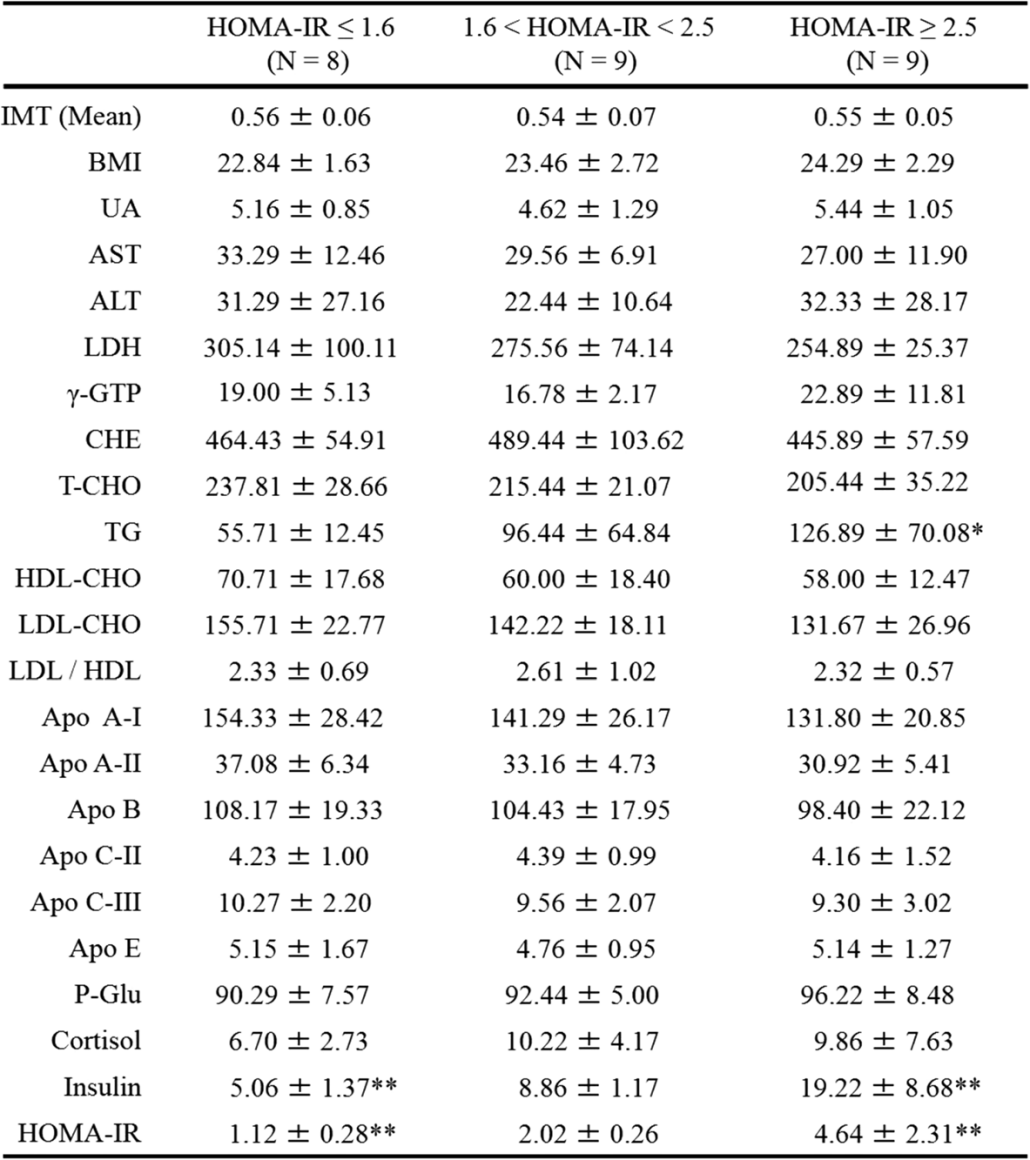
Blood test values in children with and without impaired glucose tolerance

*BMI* body mass index, *UA* uric acid, *AST* aspartate aminotransferase, *ALT* alanine aminotransferase, *LDH* lactase dehydrogenase, *γ-GTP* γ-glutamyltransferase, *CHE* colinesterase, *T-CHO* total cholesterol, *TG* triglyseride, *HDL-CHO* high-density lipoprotein cholesterol, *LDL-CHO* low-density lipoprotein cholesterol, *Apo A-I* apolipoprotein fraction A-I, *Apo A-II* apolipoprotein fraction A-II, *Apo B* apolipoprotein fraction B, *Apo C-II* apolipoprotein fraction C-II, *Apo C-III* Apolipoprotein fraction C-III, *Apo E* apolipoprotein fraction E, *P-Glu* Plasma glucose, *AST* aspartate aminotransferase, *ALT* alanine aminotransferase, *IMT* intima media thickness. Values are shown as the mean ± SD; *P < 0.05; **P < 0.01. *P* value VS a group with HOMA-IR ≤1.6

**Table 4:**
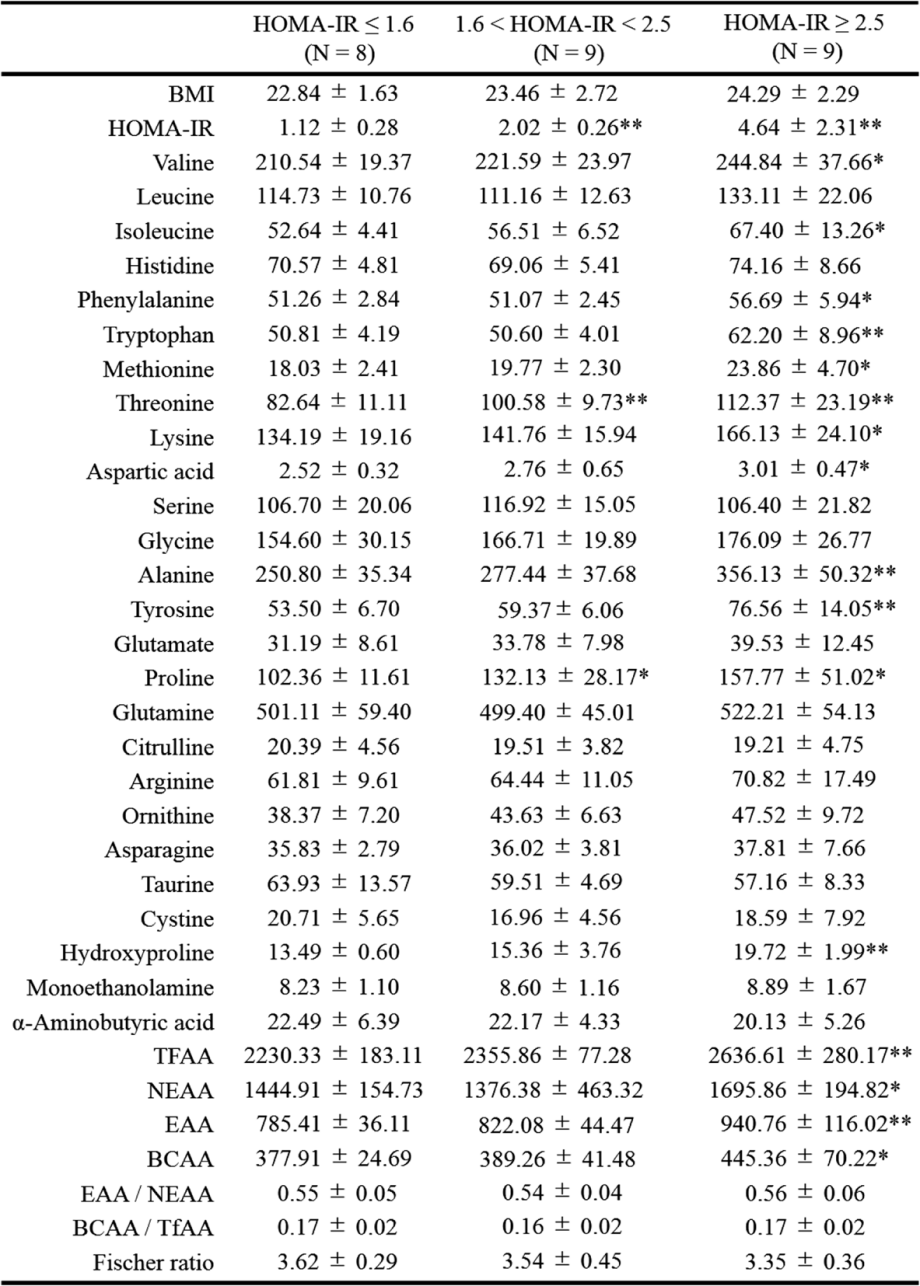
Blood amino acid levels in children with and without impaired glucose tolerance

*TFAA* total free amino acids, *EAA* essential amino acids, *NEAA* non-essential amino acids, *BCAA* branched chain amino acids, *amino acids* nmol/mL. Values are shown as mean ± SD; *P < 0.05; **P < 0.01. *P* value VS a group with HOMA-IR ≤1.6

In obese children with decreased HOMA-IR after 6 months of medication-free intervention such as nutritional and exercise guidance, the levels of Val, Leu, Ile, Asp, Ala, Tyr, Glu, and Pro decreased, but those of Gly and Ser increased (Additional file 1). In contrast, in obese children with increased HOMA-IR after intervention, all these amino acids tended to increase (Additional file 1).

### Correlations involving UA

**Fig. 3:**
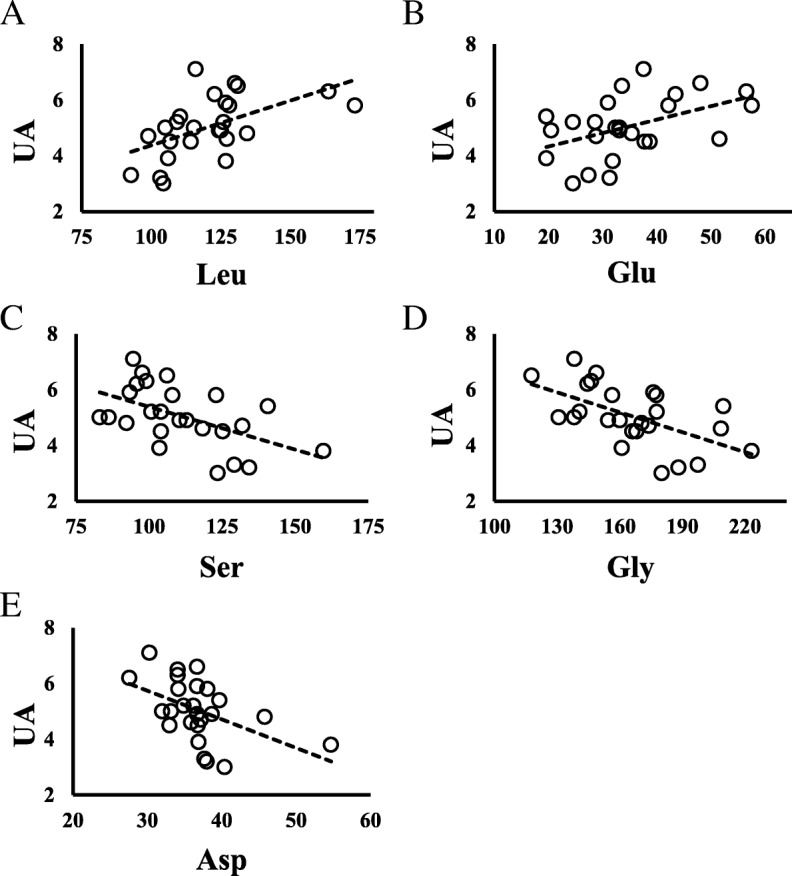
Relationships between UA and amino acids in all children. **a** UA vs Leucine; *N* = 26, y = 0.032x + 1.1692, *R*^*2*^ = 0.2925, *P* = 0.005. **b** UA vs Glutamate; *N* = 26, y = 0.048x + 3.3752, *R*^*2*^ = 0.2183, *P* = 0.019. **c** UA vs Serine; *N* = 26, y = − 0.0307x + 8.4566, *R*^*2*^ = 0.2769, *P* = 0.007. **d** UA vs Glycine; *N* = 26, y = − 0.0238x + 9.0036, *R*^*2*^ = 0.328, *P* = 0.003. **e** UA vs Asparagine; *N* = 26, y = − 0.1028x + 8.826, *R*^*2*^ = 0.2375, *P* = 0.013

Interestingly, UA was positively correlated with Leu (*P* = 0.005) and Glu (*P* = 0.019), and negatively correlated with serine (Ser), glycine (Gly), and asparagine (Asn) (*P* = 0.007, *P* = 0.003, and *P* = 0.013, respectively) (Table 2). Figure 3 shows scatter diagrams depicting these correlations. No amino acids were correlated with IMT (Table 2).

## Discussion

Table 5 summarizes the relationship between blood amino acids and HOMA-IR, UA, LDL/HDL, and IMT. Obesity often transfers from early childhood through school age, and it extends into adulthood in an estimated 50% of cases. There are some reports that obesity, hyperlipidemia, and hyperglycemia in the adult are significantly correlated with IMT, and are risk factors for severely elevated IMT [11, 12]. This correlation has also been seen in children [13, 14]. Although arteriosclerosis is not common in obese children, their IMT tends to be higher than in non-obese children [14, 15]. In this study, we found that IMT correlated negatively with HDL-CHO and positively with LDL/HDL ratio, although a positive correlation with LDL-CHO was not present. These findings suggest that obesity drives arteriosclerotic changes even in childhood. The risk factors for atherosclerosis include hypertension, hyperglycemia, and hyperlipidemia; however, few obese children develop hypertension. Hyperlipidemia and hyperglycemia are considered to be the most important risk factors for atherosclerosis. In our obese children group, blood glucose and insulin resistance did not affect IMT significantly, but the lipid metabolic parameter significantly correlated with IMT.

**Table 5:**
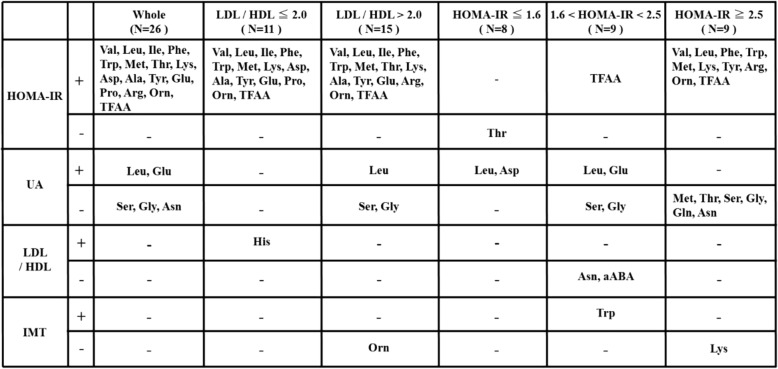
Summary of correlations between blood amino acids and HOMA-IR, UA, LDL/HDL ratio, and IMT

*Val* valine, *Leu* leucine, *Ile* isoleucine, *Phe* phenylalanine, *Trp* tryptophan, *Met* methionine, *Thr* threonine, *Lys* lysine, *Asp* asparate, *Ala* alanine, *Tyr* tyrosine, *Glu* glutamate, *Pro* proline, *Arg* arginine, *Orn* ornithine, *Ser* serine, *Gly* glycine, *Asn* asparagine, *aABA* α-aminobutyric acid

The presence in childhood of a higher number of risk factors for the development of lifestyle-related diseases is associated with greater IMT in adults [16, 17]. Raitakari et al. [16] reported that a number of risk factors for atherosclerosis measured in 12- to 18-year-old adolescents, including high levels of LDL-CHO, BMI, and systolic blood pressure, were directly related to carotid IMT in adults. The presence of these risk factors at infant and school ages also affected IMT in adults. Therefore, we should consider treating children with obesity as a disease group, rather than simply as a group with lifestyle-related risk factors for future illness.

Elevated levels of amino acids such as BCAA, Ala, Glu, Asp, and Tyr, which are related to type II diabetes, have previously been shown in obese children with HOMA-IR ≥ 2.5 [7]. We showed positive correlations between HOMA-IR and several amino acids, including TFAA, in children with impaired glucose tolerance (HOMA-IR ≥2.5). In these children, TFAA was also significantly correlated with blood glucose and insulin. When hyper-nutrition advances and impaired glucose tolerance develops, both glucose and amino acids accumulate. Cells such as hepatocytes and skeletal muscle cells become saturated, and this is considered to lead to hyper aminoacidemia.

Relevant associations between plasma amino acid levels and several other factors have also been documented. Insulin, growth hormone, glucagon, and IGF-1 play important roles in the regulation of energy metabolism in the living body [18–20], and as demonstrated in this study, insulin affects plasma amino acid levels. Some reports have demonstrated a relationship between IMT and amino acids [21, 22]. However, blood amino acids were not significantly correlated with IMT in our study. This phenomenon may be explained by a change in amino acid metabolism and insulin sensitivity in moderately obese children, because IMT was associated with LDL/HDL but not with blood insulin levels or HOMA-IR.

Associations have been shown between BCAAs and metabolic syndrome, obesity, type II diabetes, and/or insulin resistance [7, 23, 24], and BCAAs are a cardiometabolic risk marker independently of BMI category [25]. Increased plasma BCAA and lipids can lead to the development of β-cell dysfunction, which can accelerate the transition from an obese, insulin-resistant state to metabolic syndrome and type II diabetes [24]. Pozefsky et al. [26] suggested that impaired insulin activity and decreased utilization of amino acids in the muscles increased plasma BCAA levels owing to reduced uptake of BCAA in the muscles in lifestyle-related diseases. Moreover, Newgard [24] reasoned that the increased circulating blood BCAA in obese and insulin-resistant subjects partly results from a decline of AA catabolism in their adipose tissue. Readily usable glucose and lipid substrates are considered to obviate the need for AA catabolism in adipose tissue by downregulation of the BCAA catabolic enzymes through the suppression of peroxisome proliferator-activated receptor-γ signaling in such metabolic adaptations.

Another particularly relevant amino acid is Ala. Würtz et al. [27] reported that gluconeogenesis substrates including Ala increased in adults with impaired glucose tolerance. Moreover, Shimizu et al. [28] reported that depletion of plasma Ala serves as a cue to increase plasma fibroblast growth factor 21 values and enhance liver-fat communication, resulting in the activation of lipolytic genes in adipose tissues.

Amino acids are not only essential nutrients serving as an energy source for the human body, but are also involved in many biochemical processes including the biosynthesis of purines and UA production. In recent years, many factors including BMI, alcohol intake, hyperlipidemia, and diabetes have been found to contribute to increasing blood UA levels [29]. Our study indicated that in obese children, UA may be affected by amino acid metabolism rather than hyperglycemia and hyperinsulinemia. We found decreased levels of Gly and Ser with increased blood UA levels. Decreased blood Gly and Ser levels have previously been shown in adult patients with asymptomatic hyperuricemia or gout compared with healthy adult controls [30]. The same study found increased blood levels of Ala, Val, Ile, and Orn in adult patients with asymptomatic hyperuricemia, but these amino acids were not correlated with UA in our study. It seems that Gly and Ser are related to the metabolic process of increasing blood UA level [31]. Although Ser has no known relevant link with UA synthesis, Gly is needed for the de novo synthesis of purine [32], which is the biosynthetic precursor of UA. More Gly may be consumed for the biosynthesis of purine in children with obesity under hyperinsulinemia.

This study has a number of notable limitations. Principally, our sample size was relatively small, in particular with regard to comparisons between children with and without impaired glucose tolerance. This limited the statistical power to draw firm conclusions. Finally, we did not evaluate the impact of dietary and lifestyle factors, or genetic factors including family history of obesity on amino acid patterns.

In recent years, early childhood obesity prevention is required because the prevalence of overweightness and obesity in children aged 5 years and below has been increasing worldwide [33]. In Japan, examinations of physical and mental development were performed on young children at 1.5 and 3 years of age. Geserick et al. reported that most children who were obese between 2 and 6 years of age were obese in adolescence [34]. In the future, we need to perform screening and intervention for obesity at the ages of 3 and 6, prior to their entry into kindergarten and primary school, respectively. This should involve assessment of their metabolic state. It would also be desirable to study junior high school students with obesity. Analysis of metabolic profiles including amino acids in obese children and adolescents from different age groups may reveal additional problems and remedies relevant to childhood obesity.

## Conclusions

Our data support the potential of amino acid profiles as a useful marker for early intervention in childhood obesity. Importantly, these profiles reflect impaired glucose tolerance and hyperuricemia at an early obese stage. Moreover, a state of unbalanced or increased amino acids associated with obesity, such as BCAA in the blood, may exacerbate obesity and insulin sensitivity. Therefore, our results also support the view that a diet with good nutritional balance and exercise therapy that normalizes the balance of blood amino acids is important in the treatment of obesity.

## Additional file

Additional file 1: **Table S1.** (A) Blood test values in children with and without dyslipidemia. Values are given as mean ± SD; *P < 0.05; **P < 0.01. (B) Blood amino acids values in children with and without dyslipidemia. amino acid concentrations: nmol/mL. Values are given as mean ± SD; *P < 0.05; **P < 0.01. **Table S2.** Relationship between LDL/HDL ratio and IMT in children with obesity. (A) LDL/HDL vs right side IMT; *N* = 26; y = 6.3399x – 1.0341; *R*^*2*^ = 0.3125; *P* = 0.003. (B) LDL/HDL vs left side IMT; *N* = 26; y = 1.9197x + 1.334; *R*^*2*^ = 0.0312; *P* = 0.388. (C) LDL/HDL vs mean IMT; *N* = 26; y = 6.0181x – 0.8849; *R*^*2*^ = 0.1972; *P* = 0.023. **Table S3.** (A) Amino acid profiles in obese children with decreased HOMA-IR and BMI after intervention. Valine, leucine, isoleucine, alanine, and tyrosine tended to decrease, and glycine tended to increase with decreased HOMA-IR and BMI after intervention (*N* = 7). (B) Amino acid profiles in obese children with increased HOMA-IR and decreased BMI after intervention. Valine, leucine, isoleucine, phenylalanine, tryptophan, methionine, lysine, glycine, alanine, and tyrosine increased with increased HOMA-IR and decreased BMI after intervention (*N* = 14). (PPTX 88 kb)

## Data Availability

The datasets used and/or analysed during the current study are available from the corresponding author on reasonable request.

## Abbreviations

AA: Amino acid
Ala: Alanine
ALT: Alanine aminotransferase
Arg: Arginine
Asn: Asparagine
Asp: Aspartate
AST: Aspartate aminotransferase
BCAA: Branched chain amino acids
Cit: Citrulline
DM: Diabetes mellitus
EAA: Essential amino acids
Glu: Glutamate
Gly: Glycine
HDL-CHO: High-density lipoprotein cholesterol
HOMA – IR: Homeostasis model assessment-insulin resistance
Ile: Isoleucine
IMT: Intima media thickness
LDH: Lactate dehydrogenase
LDL-CHO: Low-density lipoprotein cholesterol
Leu: Leucine
Met: Methionine
Orn: Ornithine
Phe: Phenylalanine
Pro: Proline
Ser: Serine
T-CHO: Total cholesterol
TFAA: Total free amino acids
TG: Triglyceride
Trp: Tryptophan
Tyr: Tyrosine
γ-GTP: γ-glutamyltransferase
UA: Uric Acid
Val: Valine
