# Supplementary material for: Associations among amino acid, lipid, and glucose metabolic profiles in childhood obesity
Source: BMC Pediatr. 2019 Aug 6;19:273. doi: 10.1186/s12887-019-1647-8 (PMC6683574; doi:10.1186/s12887-019-1647-8)
Supplement: Supplementary file 1 — Table S1. (A) Blood test values in children with and without dyslipidemia. Values are given as mean ± SD; *P < 0.05; **P < 0.01. (B) Blood amino acids values in children with and without dyslipidemia. amino acid concentrations: nmol/mL. Values are given as mean ± SD; *P < 0.05; **P < 0.01. Table S2. Relationship between LDL/HDL ratio and IMT in children with obesity. (A) LDL/HDL vs right side IMT; N = 26; y = 6.3399x – 1.0341; R2 = 0.3125; P = 0.003. (B) LDL/HDL vs left side IMT; N = 26; y = 1.9197x + 1.334; R2 = 0.0312; P = 0.388. (C) LDL/HDL vs mean IMT; N = 26; y = 6.0181x – 0.8849; R2 = 0.1972; P = 0.023. Table S3. (A) Amino acid profiles in obese children with decreased HOMA-IR and BMI after intervention. Valine, leucine, isoleucine, alanine, and tyrosine tended to decrease, and glycine tended to increase with decreased HOMA-IR and BMI after intervention (N = 7). (B) Amino acid profiles in obese children with increased HOMA-IR and decreased BMI after intervention. Valine, leucine, isoleucine, phenylalanine, tryptophan, methionine, lysine, glycine, alanine, and tyrosine increased with increased HOMA-IR and decreased BMI after intervention (N = 14). (PPTX 88 kb) [file 12887_2019_1647_MOESM1_ESM.pptx]

## Slide 1
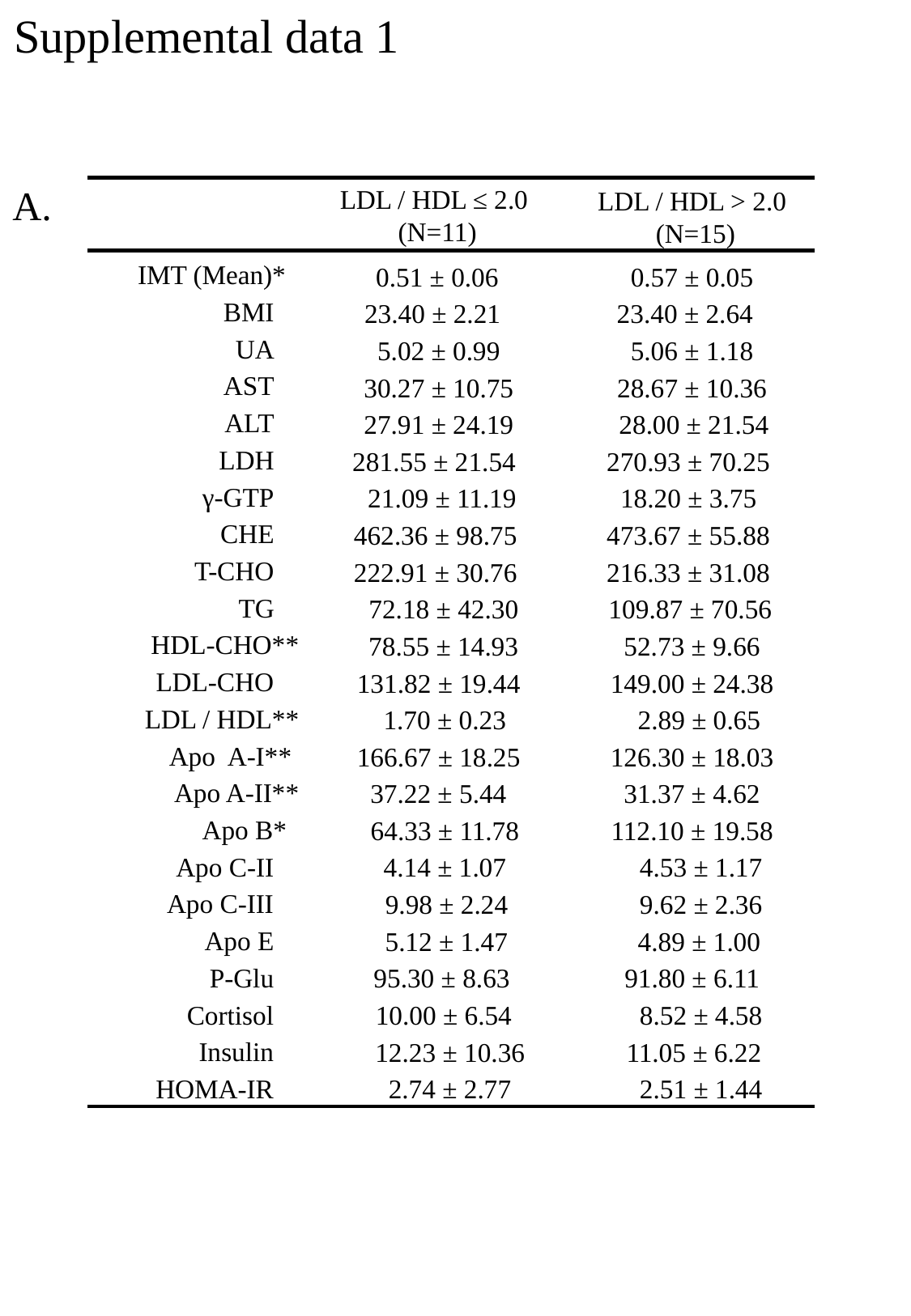

Supplemental data 1
A.
LDL / HDL ≤ 2.0
(N=11)
0.51 ± 0.06
23.40 ± 2.21
5.02 ± 0.99
30.27 ± 10.75
27.91 ± 24.19
281.55 ± 21.54
21.09 ± 11.19
462.36 ± 98.75
222.91 ± 30.76
72.18 ± 42.30
78.55 ± 14.93
131.82 ± 19.44
1.70 ± 0.23
166.67 ± 18.25
37.22 ± 5.44
64.33 ± 11.78
4.14 ± 1.07
9.98 ± 2.24
5.12 ± 1.47
95.30 ± 8.63
10.00 ± 6.54
12.23 ± 10.36
2.74 ± 2.77
LDL / HDL > 2.0
 (N=15)
0.57 ± 0.05
23.40 ± 2.64
5.06 ± 1.18
28.67 ± 10.36
28.00 ± 21.54
270.93 ± 70.25
18.20 ± 3.75
473.67 ± 55.88
216.33 ± 31.08
109.87 ± 70.56
52.73 ± 9.66
149.00 ± 24.38
2.89 ± 0.65
126.30 ± 18.03
31.37 ± 4.62
112.10 ± 19.58
4.53 ± 1.17
9.62 ± 2.36
4.89 ± 1.00
91.80 ± 6.11
8.52 ± 4.58
11.05 ± 6.22
2.51 ± 1.44
IMT (Mean)*
BMI
UA
AST
ALT
LDH
γ-GTP
CHE
T-CHO
TG
HDL-CHO**
LDL-CHO
LDL / HDL**
Apo A-I**
Apo A-II**
Apo B*
Apo C-II
Apo C-III
Apo E
P-Glu
Cortisol
Insulin
HOMA-IR

## Slide 2
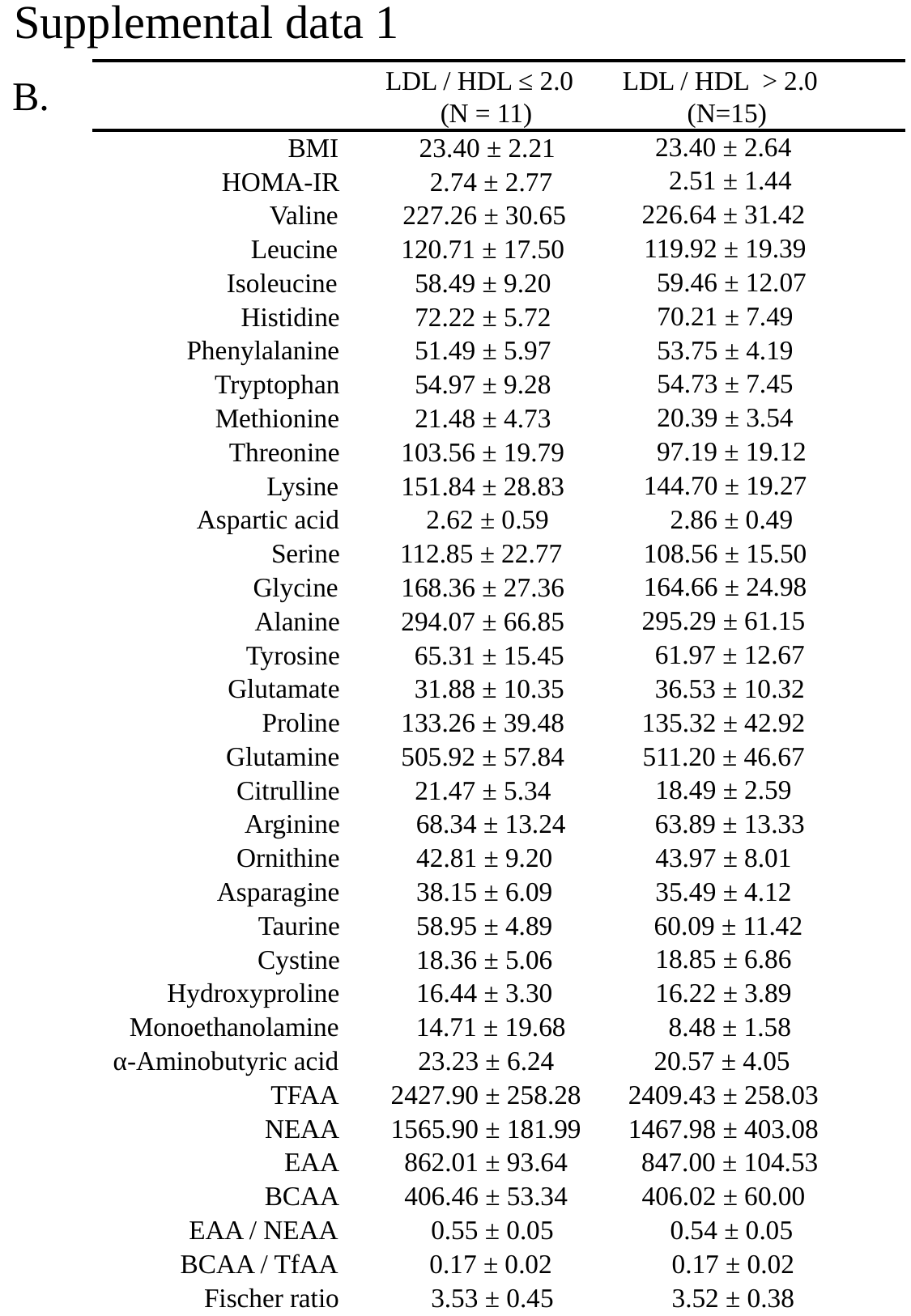

Supplemental data 1
LDL / HDL ≤ 2.0
 (N = 11)
LDL / HDL > 2.0
 (N=15)
23.40 ± 2.64
BMI
23.40 ± 2.21
2.51 ± 1.44
HOMA-IR
2.74 ± 2.77
226.64 ± 31.42
Valine
227.26 ± 30.65
119.92 ± 19.39
Leucine
120.71 ± 17.50
59.46 ± 12.07
Isoleucine
58.49 ± 9.20
70.21 ± 7.49
Histidine
72.22 ± 5.72
53.75 ± 4.19
Phenylalanine
51.49 ± 5.97
54.73 ± 7.45
Tryptophan
54.97 ± 9.28
20.39 ± 3.54
Methionine
21.48 ± 4.73
97.19 ± 19.12
Threonine
103.56 ± 19.79
144.70 ± 19.27
Lysine
151.84 ± 28.83
2.86 ± 0.49
Aspartic acid
2.62 ± 0.59
108.56 ± 15.50
Serine
112.85 ± 22.77
164.66 ± 24.98
Glycine
168.36 ± 27.36
295.29 ± 61.15
Alanine
294.07 ± 66.85
61.97 ± 12.67
Tyrosine
65.31 ± 15.45
36.53 ± 10.32
Glutamate
31.88 ± 10.35
135.32 ± 42.92
Proline
133.26 ± 39.48
511.20 ± 46.67
Glutamine
505.92 ± 57.84
18.49 ± 2.59
Citrulline
21.47 ± 5.34
63.89 ± 13.33
Arginine
68.34 ± 13.24
43.97 ± 8.01
Ornithine
42.81 ± 9.20
35.49 ± 4.12
Asparagine
38.15 ± 6.09
60.09 ± 11.42
Taurine
58.95 ± 4.89
18.85 ± 6.86
Cystine
18.36 ± 5.06
16.22 ± 3.89
Hydroxyproline
16.44 ± 3.30
8.48 ± 1.58
Monoethanolamine
14.71 ± 19.68
20.57 ± 4.05
α-Aminobutyric acid
23.23 ± 6.24
2409.43 ± 258.03
 TFAA
2427.90 ± 258.28
1467.98 ± 403.08
NEAA
1565.90 ± 181.99
847.00 ± 104.53
EAA
862.01 ± 93.64
406.02 ± 60.00
BCAA
406.46 ± 53.34
0.54 ± 0.05
EAA / NEAA
0.55 ± 0.05
0.17 ± 0.02
BCAA / TfAA
0.17 ± 0.02
Fischer ratio
3.53 ± 0.45
3.52 ± 0.38
B.

## Slide 3
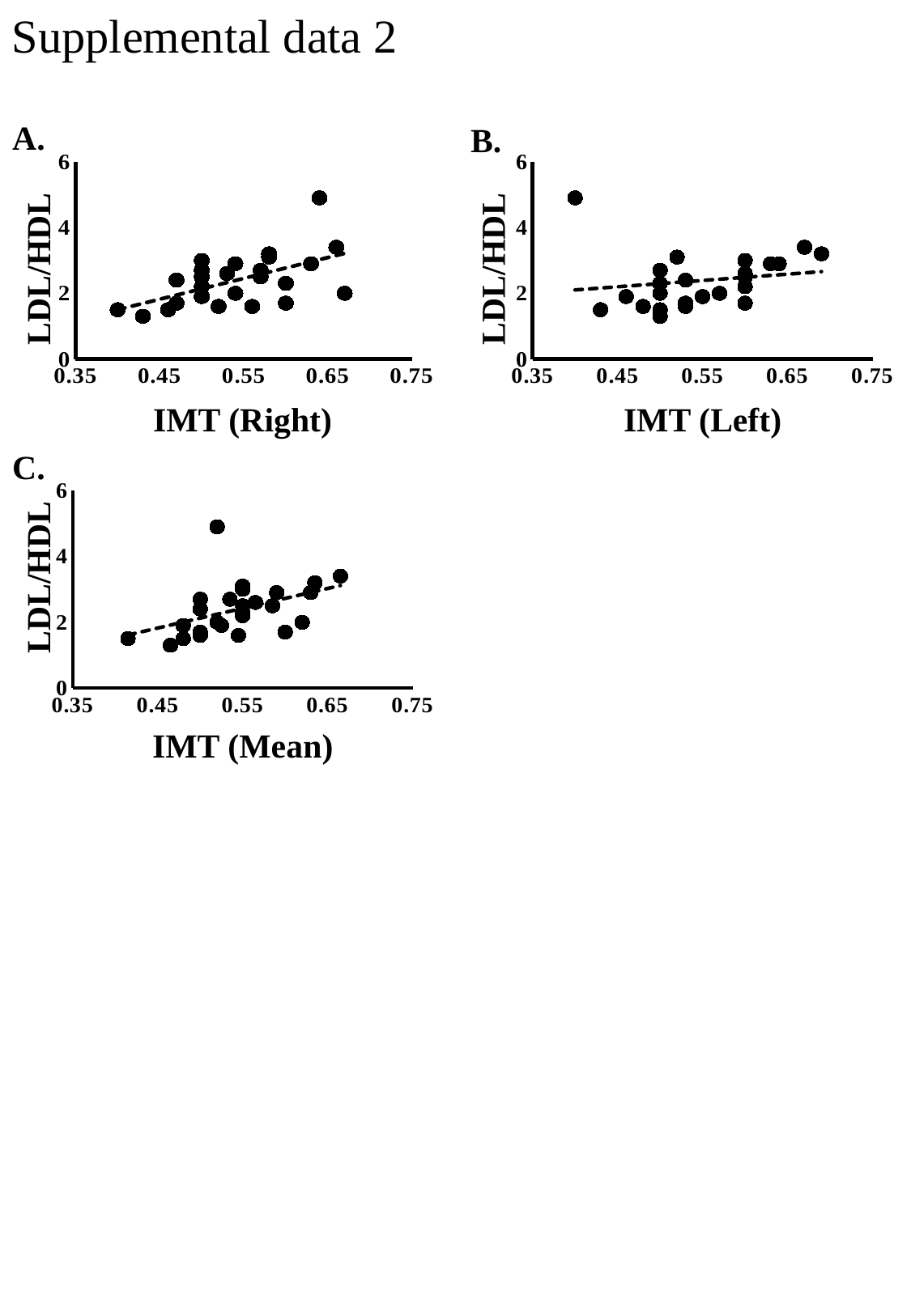

Supplemental data 2
A.
B.
### Chart
| Category | IMT-rt (mm) |
|---|---|
### Chart
| Category | IMT-lt (mm) |
|---|---|LDL/HDL
LDL/HDL
IMT (Right)
IMT (Left)
C.
### Chart
| Category | IMT (Ave) |
|---|---|LDL/HDL
IMT (Mean)

## Slide 4
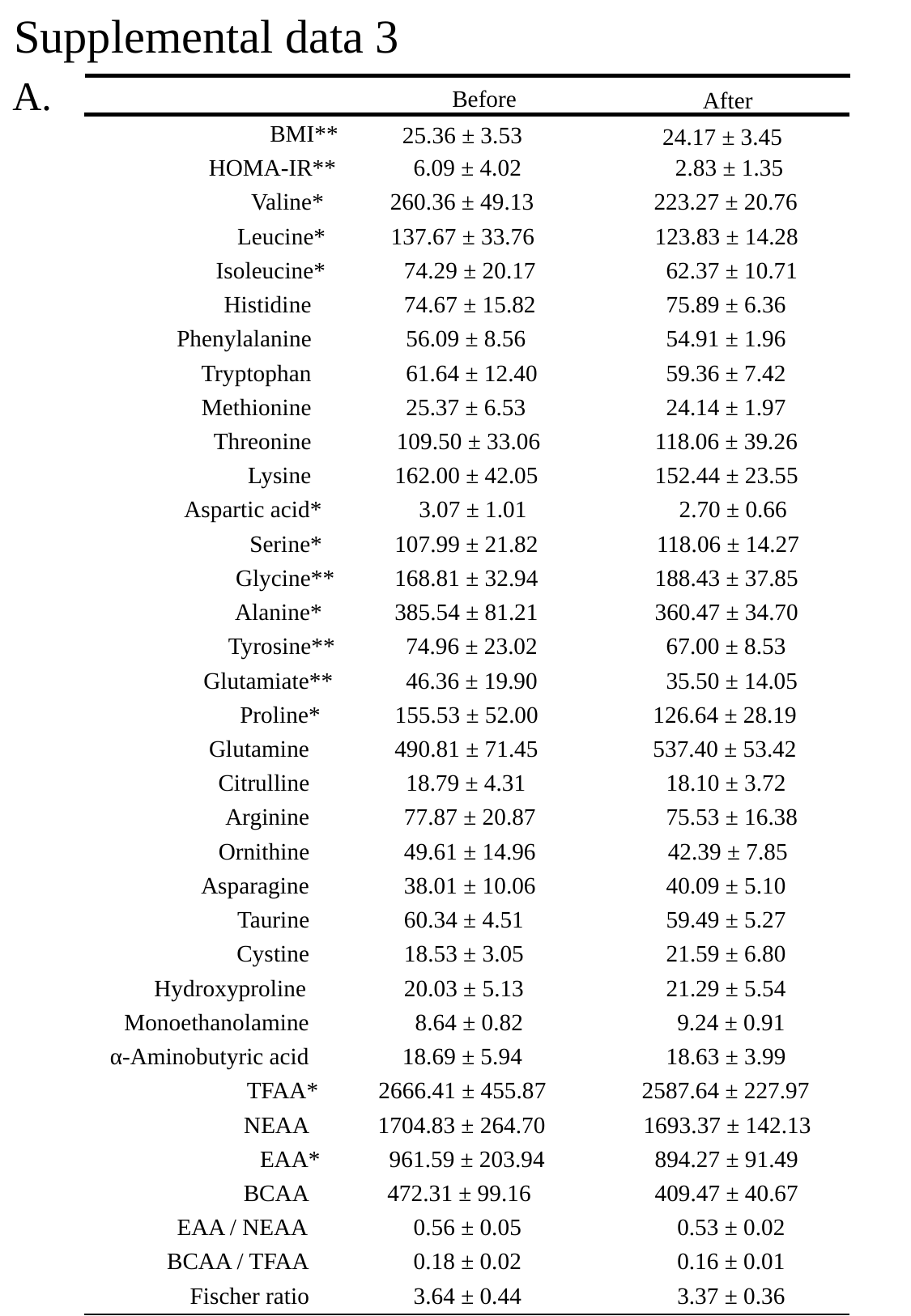

Supplemental data 3
A.
Before
After
BMI**
25.36 ± 3.53
24.17 ± 3.45
HOMA-IR**
6.09 ± 4.02
2.83 ± 1.35
Valine*
260.36 ± 49.13
223.27 ± 20.76
Leucine*
137.67 ± 33.76
123.83 ± 14.28
Isoleucine*
74.29 ± 20.17
62.37 ± 10.71
Histidine
74.67 ± 15.82
75.89 ± 6.36
Phenylalanine
56.09 ± 8.56
54.91 ± 1.96
Tryptophan
61.64 ± 12.40
59.36 ± 7.42
Methionine
25.37 ± 6.53
24.14 ± 1.97
Threonine
109.50 ± 33.06
118.06 ± 39.26
Lysine
162.00 ± 42.05
152.44 ± 23.55
Aspartic acid*
3.07 ± 1.01
2.70 ± 0.66
Serine*
107.99 ± 21.82
118.06 ± 14.27
Glycine**
168.81 ± 32.94
188.43 ± 37.85
Alanine*
385.54 ± 81.21
360.47 ± 34.70
Tyrosine**
74.96 ± 23.02
67.00 ± 8.53
Glutamiate**
46.36 ± 19.90
35.50 ± 14.05
Proline*
155.53 ± 52.00
126.64 ± 28.19
Glutamine
490.81 ± 71.45
537.40 ± 53.42
Citrulline
18.79 ± 4.31
18.10 ± 3.72
Arginine
77.87 ± 20.87
75.53 ± 16.38
Ornithine
49.61 ± 14.96
42.39 ± 7.85
Asparagine
38.01 ± 10.06
40.09 ± 5.10
Taurine
60.34 ± 4.51
59.49 ± 5.27
Cystine
18.53 ± 3.05
21.59 ± 6.80
Hydroxyproline
20.03 ± 5.13
21.29 ± 5.54
Monoethanolamine
8.64 ± 0.82
9.24 ± 0.91
α-Aminobutyric acid
18.69 ± 5.94
18.63 ± 3.99
2666.41 ± 455.87
TFAA*
2587.64 ± 227.97
NEAA
1704.83 ± 264.70
1693.37 ± 142.13
EAA*
961.59 ± 203.94
894.27 ± 91.49
BCAA
472.31 ± 99.16
409.47 ± 40.67
EAA / NEAA
0.56 ± 0.05
0.53 ± 0.02
BCAA / TFAA
0.18 ± 0.02
0.16 ± 0.01
Fischer ratio
3.64 ± 0.44
3.37 ± 0.36

## Slide 5
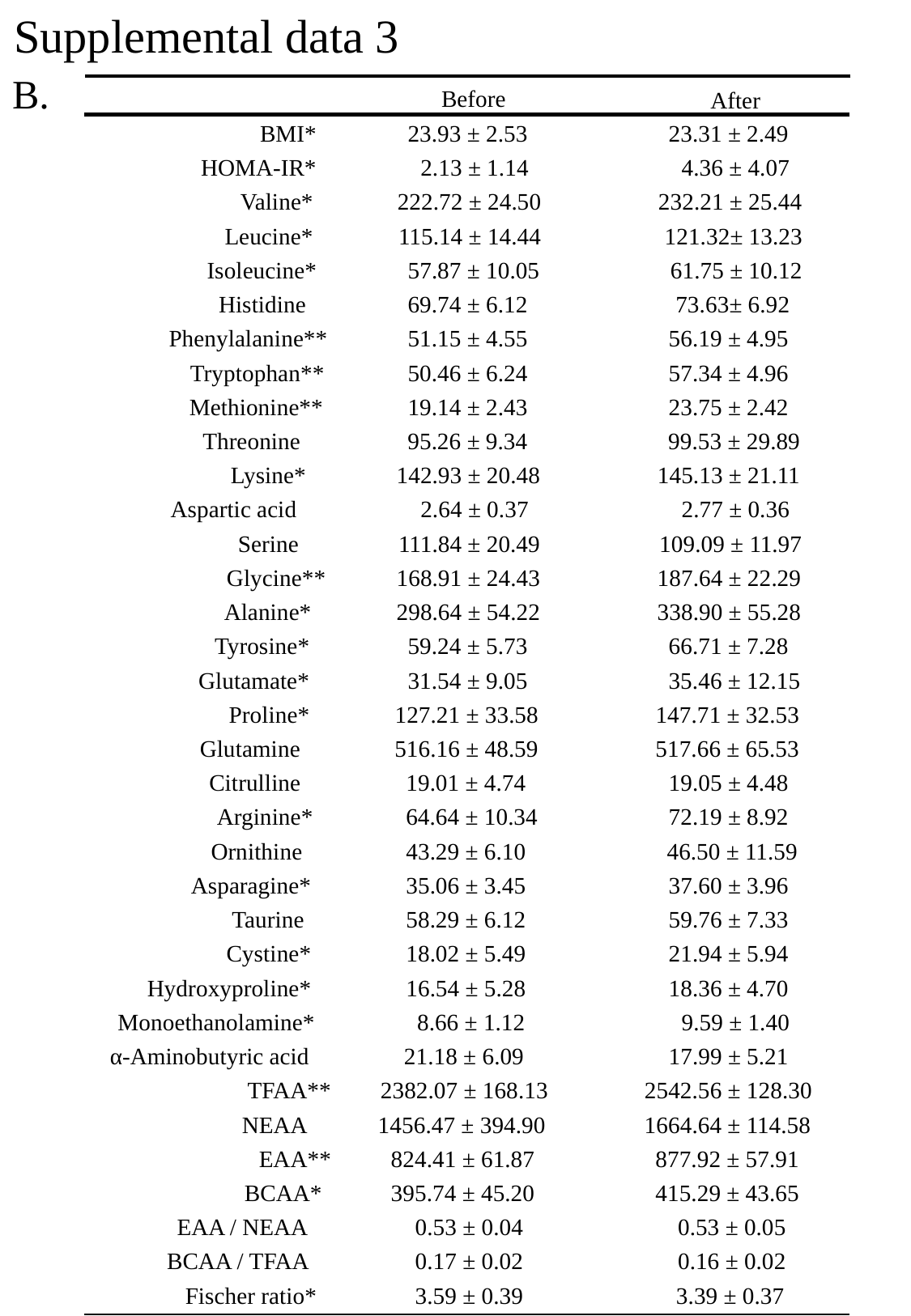

Supplemental data 3
B.
Before
After
BMI*
23.93 ± 2.53
23.31 ± 2.49
HOMA-IR*
2.13 ± 1.14
4.36 ± 4.07
Valine*
222.72 ± 24.50
232.21 ± 25.44
Leucine*
115.14 ± 14.44
121.32± 13.23
Isoleucine*
57.87 ± 10.05
61.75 ± 10.12
Histidine
69.74 ± 6.12
73.63± 6.92
Phenylalanine**
51.15 ± 4.55
56.19 ± 4.95
Tryptophan**
50.46 ± 6.24
57.34 ± 4.96
Methionine**
19.14 ± 2.43
23.75 ± 2.42
Threonine
95.26 ± 9.34
99.53 ± 29.89
Lysine*
142.93 ± 20.48
145.13 ± 21.11
Aspartic acid
2.64 ± 0.37
2.77 ± 0.36
Serine
111.84 ± 20.49
109.09 ± 11.97
Glycine**
168.91 ± 24.43
187.64 ± 22.29
Alanine*
298.64 ± 54.22
338.90 ± 55.28
Tyrosine*
59.24 ± 5.73
66.71 ± 7.28
Glutamate*
31.54 ± 9.05
35.46 ± 12.15
Proline*
127.21 ± 33.58
147.71 ± 32.53
Glutamine
516.16 ± 48.59
517.66 ± 65.53
Citrulline
19.01 ± 4.74
19.05 ± 4.48
Arginine*
64.64 ± 10.34
72.19 ± 8.92
Ornithine
43.29 ± 6.10
46.50 ± 11.59
Asparagine*
35.06 ± 3.45
37.60 ± 3.96
Taurine
58.29 ± 6.12
59.76 ± 7.33
Cystine*
18.02 ± 5.49
21.94 ± 5.94
Hydroxyproline*
16.54 ± 5.28
18.36 ± 4.70
Monoethanolamine*
8.66 ± 1.12
9.59 ± 1.40
α-Aminobutyric acid
21.18 ± 6.09
17.99 ± 5.21
2382.07 ± 168.13
TFAA**
2542.56 ± 128.30
NEAA
1456.47 ± 394.90
1664.64 ± 114.58
EAA**
824.41 ± 61.87
877.92 ± 57.91
BCAA*
395.74 ± 45.20
415.29 ± 43.65
EAA / NEAA
0.53 ± 0.04
0.53 ± 0.05
BCAA / TFAA
0.17 ± 0.02
0.16 ± 0.02
Fischer ratio*
3.59 ± 0.39
3.39 ± 0.37
